# Products of gut microbial Toll/interleukin-1 receptor domain NADase activities in gnotobiotic mice and Bangladeshi children with malnutrition

**DOI:** 10.1016/j.celrep.2022.110738

**Published:** 2022-04-26

**Authors:** James S. Weagley, Mark Zaydman, Siddarth Venkatesh, Yo Sasaki, Neha Damaraju, Alex Yenkin, William Buchser, Dmitry A. Rodionov, Andrei Osterman, Tahmeed Ahmed, Michael J. Barratt, Aaron DiAntonio, Jeffrey Milbrandt, Jeffrey I. Gordon

**Affiliations:** 1 Edison Family Center for Genome Sciences and Systems Biology, Washington University School of Medicine, St. Louis, MO 63110, USA; 2 Center for Gut Microbiome and Nutrition Research, Washington University School of Medicine, St. Louis, MO 63110, USA; 3 Department of Pathology and Immunology, Washington University School of Medicine, St. Louis, MO 63110, USA; 4 Department of Genetics, Washington University School of Medicine, St. Louis, MO 63110, USA; 5 Department of Developmental Biology, Washington University School of Medicine, St. Louis, MO 63110, USA; 6 Infectious and Inflammatory Disease Center, Sanford Burnham Prebys Medical Discovery Institute, La Jolla, CA 92037, USA; 7 A.A. Kharkevich Institute for Information Transmission Problems, Russian Academy of Sciences, Moscow 127994, Russia; 8 International Centre for Diarrhoeal Disease Research, Bangladesh (icddr,b), Dhaka 1212, Bangladesh; 9 Hope Center for Neurological Disorders, Washington University School of Medicine, St. Louis, MO 63110, USA

**Keywords:** NAD metabolism, TIR domain structure/activity relationships, human gut microbiome development/functional profiling, gnotobiotic mice, defined microbial communities, childhood malnutrition

## Abstract

Perturbed gut microbiome development has been linked to childhood malnutrition. Here, we characterize bacterial Toll/interleukin-1 receptor (TIR) protein domains that metabolize nicotinamide adenine dinucleotide (NAD), a co-enzyme with far-reaching effects on human physiology. A consortium of 26 human gut bacterial strains, representing the diversity of TIRs observed in the microbiome and the NAD hydrolase (NADase) activities of a subset of 152 bacterial TIRs assayed *in vitro*, was introduced into germ-free mice. Integrating mass spectrometry and microbial RNA sequencing (RNA-seq) with consortium membership manipulation disclosed that a variant of cyclic-ADPR (v-cADPR-x) is a specific product of TIR NADase activity and a prominent, colonization-discriminatory, taxon-specific metabolite. Guided by bioinformatic analyses of biochemically validated TIRs, we find that acute malnutrition is associated with decreased fecal levels of genes encoding TIRs known or predicted to generate v-cADPR-x, as well as decreased levels of the metabolite itself. These results underscore the need to consider microbiome TIR NADases when evaluating NAD metabolism in the human holobiont.

## Highlights

• Phylogenetically diverse human gut bacteria encode TIR-domain-containing proteins
• *In vitro* assays of >150 human gut bacterial TIRs disclose diverse NADase activities
• v-cADPR-x-TIR is prominently expressed *in vivo* by a model human gut microbiome
• Microbiome TIR NADase content is altered in children with malnutrition

## Graphical abstract

**Figure:**
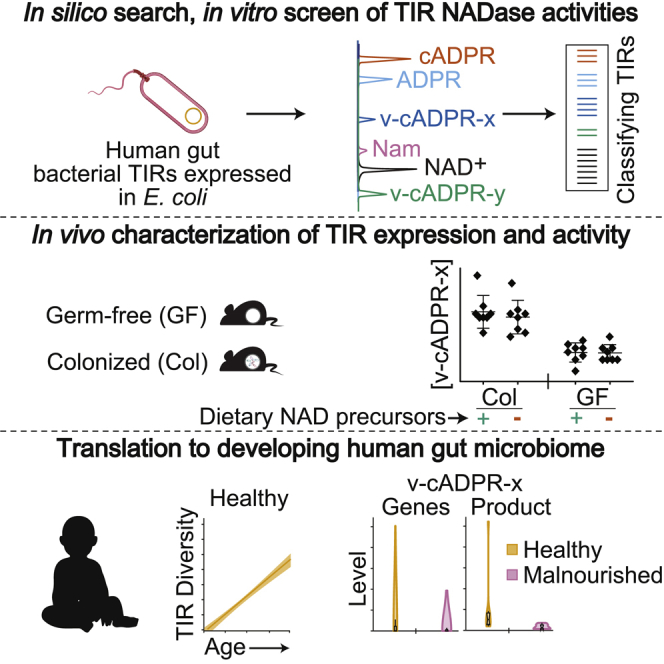

## Teaser

Weagley et al. characterize NAD metabolic activities of TIR domains in human gut bacterial proteins *in vitro* and in gnotobiotic mice. They show that TIR representation and activities differ in the developing microbiomes of age-matched healthy Bangladeshi infants and children versus those with malnutrition.

## Introduction

Studies of healthy members of birth cohorts from low- and middle-income countries have disclosed features of a program of gut microbial community assembly that is normally completed by the end of the 2^nd^ postnatal year. This process is disrupted in children with moderate acute malnutrition (MAM) and severe acute malnutrition (SAM), leaving them with immature communities (Subramanian et al., 2014; Raman et al., 2019). Preclinical studies indicate that disrupted microbiota development is a contributing cause and not simply an effect of malnutrition (Gehrig et al., 2019). This notion is supported by the results of a randomized controlled clinical study of a microbiota-directed complementary food (MDCF) formulation and a commonly employed ready-to-use supplementary food (RUSF) in Bangladeshi children with MAM. The MDCF produced a statistically significant greater degree of microbiota repair, change in plasma proteomic mediators and biomarkers of musculoskeletal, neurodevelopmental, and immune function, and improved ponderal growth compared with the calorically denser RUSF (Chen et al., 2021).

Nicotinamide adenine dinucleotide (NAD) is an essential co-factor for myriad enzymes involved in metabolic reactions affecting all mammalian cell types. Host catabolism of tryptophan through the kynurenine pathway produces several bioactive molecules, including NAD (Kennedy et al., 2017). Dietary deficiency of tryptophan is common in children with undernutrition and has been linked to impaired growth (Kosek et al., 2016). The term “niacin” (vitamin B3) traditionally refers to both nicotinamide (Nam) and nicotinic acid (NA)—two of the primary precursors of NAD in humans. Insufficient vitamin B3 or tryptophan in the diet is the cause of pellagra, a disease endemic in many countries with undernutrition (WHO, 2002). In addition, NAD metabolism is a key regulator of intestinal inflammation (Gerner et al., 2018). Intriguingly, comparing the gut microbiomes of healthy Bangladeshi infants and children with those who have SAM revealed that the SAM microbiome had reduced proportional representation of several age-discriminatory metabolic pathways, including those involved in niacin/NAD phosphate (NADP) biosynthesis (Gehrig et al., 2019).

**Figure 1:**
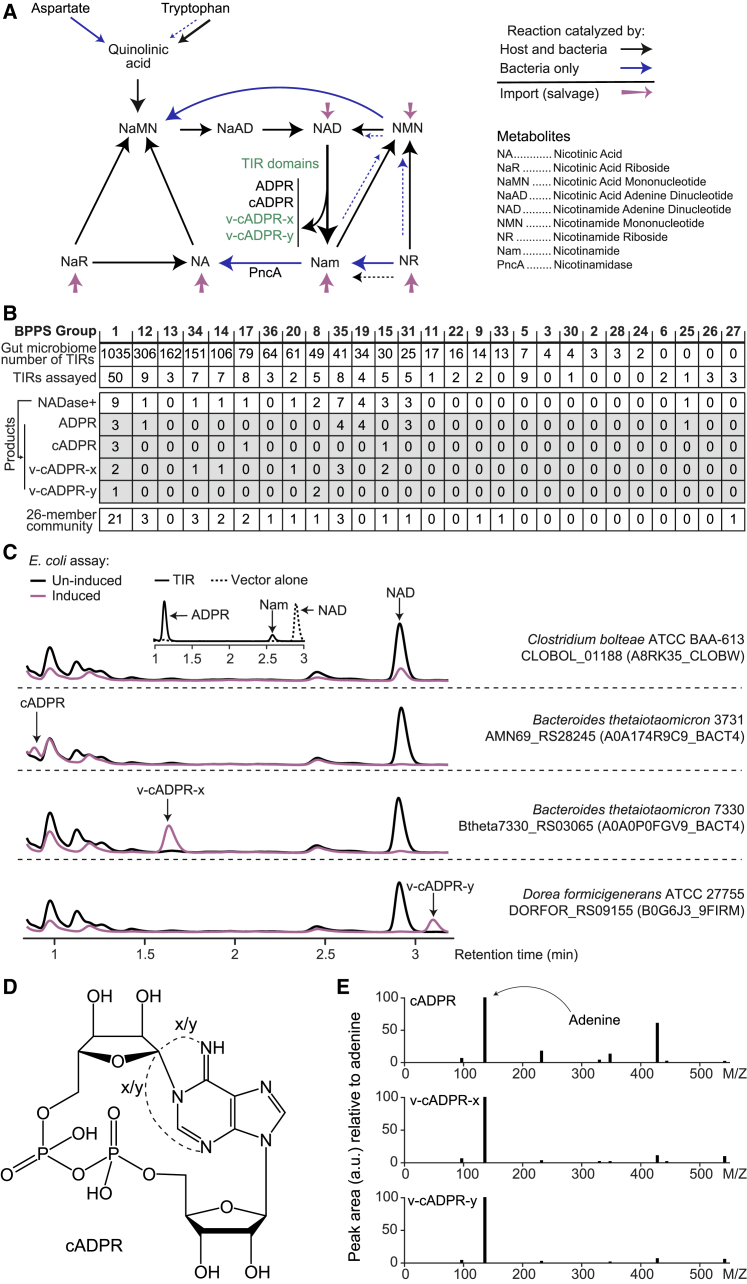
*In vitro* assays of NADase activities associated with TIR domains (A) Overview of NAD metabolism in humans and bacteria. The predominant pathways catalyzed by bacteria are shown with a solid blue line, while the pathways predominantly utilized by humans are denoted with a solid black line. Reactions that are known, but not widely distributed in bacteria, are indicated with blue dashed lines. (B) Summary of the number of TIR domains assigned to groups defined by Bayesian partitioning with pattern selections (BPPS). The left to right order of BPPS groups is based on the number of TIRs in that group that were identified in 278 fecal microbiome samples obtained from 30 healthy Bangladeshi children and 14 children with acute malnutrition. Subsequent rows indicate the number of TIRs in a given BPPS group that were assayed for NADase activity *in vitro* after expression in *E. coli* and the products that they generated from NAD (ADPR, cADPR, v-cADPR-x, or v-cADPR-y). The last row describes the number of TIRs identified in the proteomes of the 26 human gut bacterial strains that were introduced into gnotobiotic mice. (C) Examples of results of HPLC-based assays of NADase activity in recombinant TIRs produced by the isopropyl β-D-1-thiogalactopyranoside (IPTG)-inducible *E. coli* expression system. Peaks corresponding to cADPR and its variants are labeled. The inset in the top panel shows production of ADPR and Nam from the purified *C. bolteae* CLOBOL_01188 protein. (D) Structure of cADPR (Lee et al., 1994). The proposed cyclization sites of its two variant forms, v-cADPR-x and v-cADPR-y, are denoted by dashed lines. (E) MS/MS fragmentation pattern of cADPR, v-cADPR-x, and v-cADPR-y, with the peak area (absorbance units; a.u.) of each fragment normalized to the adenine moiety (m/z 136), which had the largest peak area.

Most microbes that produce NAD and its precursors utilize aspartate as the initial substrate for *de novo* biosynthesis (Figure 1A; Brenner, 2005; Gazzaniga et al., 2009). In the canonical pathway for microbes, aspartate is converted to iminoaspartate via L-aspartate oxidase or aspartate dehydrogenase and then to quinolinic acid via quinolinate synthase (Gazzaniga et al., 2009). Quinolinic acid is then converted to nicotinic acid mononucleotide (NaMN) by nicotinate-nucleotide pyrophosphorylase in a manner homologous to the reaction catalyzed by quinolinate phosphoribosyltransferase in mammals (Bogan and Brenner, 2008; Gazzaniga et al., 2009; Terakata et al., 2012) (Figure 1A). Both the host and most microbes can salvage NAD precursors from their environments as well; the precursors that can be salvaged differ among microbes, host tissues, and stage of host development (Bogan and Brenner, 2008; Boshoff et al., 2008; Sorci et al., 2013; Figure 1A).

NAD metabolism by gut microbes is intertwined with host NAD metabolism. Recent work has documented how bacterial nicotinamidase activity can alter host NAD metabolism. The mechanism involves bacterial nicotinamidase (PncA)-catalyzed conversion of nicotinamide to nicotinic acid, a deamidated precursor of NAD that is readily salvaged by the host through the Preiss-Handler pathway (Preiss and Handler, 1958; Shats et al., 2020). Another example is provided by bacterially encoded ADP-ribosyltransferases (ADPRTs); a recent report (Brown et al., 2021) indicated that bacteria express ADPRTs, which can utilize NAD to transfer ADP-ribose (ADPR) onto host proteins, stimulating release of inosine, a carbon source that is accessible to these bacteria. Furthermore, human gut microbial NAD metabolism has the capacity to modify pathologic processes, including certain forms of neurodegeneration (e.g., Blacher et al., 2019).

In the current study, we explore the relationship between malnutrition and bacterial metabolism of NAD via another potential trans-kingdom route—one involving enzymatically active Toll/interleukin-1 receptor (TIR) domains present in human gut bacterial proteomes.

Eukaryotic TIR domains were first shown to mediate protein-protein interactions in innate immune signaling pathways (Xu et al., 2000). These domains were subsequently found to possess NAD hydrolase (NADase) activity utilizing NAD as a substrate to generate Nam and ADPR-like products. This activity was initially described in the TIR domain of SARM1 and is a critical mediator of the degeneration that follows axonal injury (Essuman et al., 2017; Gerdts et al., 2015). TIR domains have also been identified in plant proteins that degrade NAD in responses to pathogen recognition that lead to cell death (Horsefield et al., 2019; Wan et al., 2019).

Most bacterial TIR domains and non-TIR NADases have been characterized in pathogens, where they play a role in mediating virulence, including evasion of host immune responses (Essuman et al., 2018; Bricker et al., 2005; Coronas-Serna et al., 2020; McGuire and Arthur, 2015; Tak et al., 2019). In addition, bacterial NADases function as effectors in type VI and type VII secretion systems involved in inter-bacterial antagonism (Fridman et al., 2020; Klein et al., 2018; Tang et al., 2018; Whitney et al., 2015). TIR domains are also present in non-pathogenic bacteria, where they appear in proteins with diverse domain architectures and exhibit high levels of primary sequence heterogeneity (Toshchakov and Neuwald, 2020). Comparative genomic approaches identified a relationship between TIR domains and nucleotide sensing and signaling domains in bacterial proteomes (Burroughs et al., 2015). Similar approaches found TIR domains enriched in islands of phage defense genes and identified them as essential components of retron-associated phage defense systems (Gao et al., 2020; Millman et al., 2020), the “Thoeris” defense system (Doron et al., 2018; Ka et al., 2020; Lopatina at al., 2020), cyclic nucleotide-based anti-phage signaling systems (CBASSs) (Cohen et al., 2019; Tal et al., 2021), and other bacterial immune systems (Burroughs and Aravind, 2020). Since bacteria differ in their biosynthetic, salvage, and metabolic capabilities related to vitamin B3, the availability of NAD and its precursors may have differential effects on their fitness (Rodionov et al., 2019).

There have been limited studies of the biochemical activities of bacterial TIR domains; the information available is principally derived from pathogens and *in vitro* assays rather than *in vivo* analyses. In the present report, we use *in vitro* biochemical assays to first characterize the NADase activities and metabolic products of 152 TIR domains present in proteins encoded in the genomes of phylogenetically diverse bacterial taxa and in the developing microbiomes of Bangladeshi infants and children. In follow-up *in vivo* studies, gnotobiotic mice were colonized with a 26-member consortium of human gut bacterial strains encoding 42 proteins containing a total of 44 TIR domains representing the diversity of TIRs observed in the microbiome and NAD enzymatic activities observed *in vitro*. TIR expression and NAD metabolism were defined in these animals under conditions where dietary NAD precursor availability was deliberately varied. Differences between mice colonized with the 26-member consortium and germ-free animals were pronounced when measuring cecal levels of an isomeric variant of cyclic ADP-ribose (v-cADPR-x)—a product of TIR NADase activity observed in our *in vitro* assay. Follow-up colonization experiments in which subsets of the bacterial consortium were introduced into gnotobiotic mice revealed that an NADase-positive TIR domain in *Bacteroides xylanisolvens* was the predominant source of v-cADPR-x. The results of our *in vitro* screen provided a means for evaluating the representation of this and other classes of TIRs in the developing microbiomes of Bangladeshi children. These preclinical *in vitro* and *in vivo* studies, combined with their translation to a small cohort of infants and children indicate that one manifestation of disrupted gut microbiome development in children with malnutrition is perturbed NAD metabolism involving enzymatically active TIR domains represented in the proteomes of resident bacteria.

## Results

### Constructing a model human gut microbial community with representative TIR domains

To characterize gut bacterial TIR NADase activity *in vivo*, we sought to assemble a collection of cultured human gut bacterial species based on the following criteria: (1) they are well represented in the developing and/or adult human gut microbiota, (2) they are known to be successful colonizers of the intestines of germ-free animals, and (3) they contain proteins that include TIR domains that are well represented in the developing microbial communities of infants and children. This latter criterion was informed by an analysis of TIR domains in the gut microbiomes of Bangladeshi infants and children. To conduct this analysis, we annotated TIR domains in a dataset generated by shotgun sequencing of fecal samples from Bangladeshi infants and children who resided in a densely populated urban slum (Mirpur) located in one of the districts of Dhaka; 212 of these biospecimens were obtained by serial sampling of 30 members of a birth cohort with consistently healthy growth phenotypes (as defined by serial anthropometry), while 66 samples were from 14 children who presented at 7–16 months of age with SAM and were then followed for 12 months after nutritional rehabilitation (Gehrig et al., 2019).

We used a hidden Markov model (HMM)-based approach to identify TIR domains in proteins encoded by the microbiomes of these infants and children. TIR domains were defined as present if they exhibited significant similarity to members of the six Pfam families that encompass known TIR domains (El-Gebali et al., 2019; http://hmmer.org/; see STAR Methods for details). Toshchakov and Neuwald (2020) recently applied Bayesian partitioning and pattern selection (BPPS) to a large collection of publicly available TIR domains to create an additional approach for categorizing their sequence and structural diversity; the results yielded 36 BPPS groups. TIR domains identified in the sampled microbiomes using the HMM-based approach were assigned to BPPS groups using Diamond (Buchfink et al., 2021; Toshchakov and Neuwald, 2020). The results are summarized in Figure 1B. A total of 3,072 TIR domains were found among the 17,343,370 open reading frames (ORFs) identified in all sampled microbiomes (2,504 TIRs from 2,491 ORFs among the 13,976,979 ORFs in the 212 microbiome samples from healthy children; 568 TIRs from 566 ORFs among the 3,366,391 ORFs identified in the 66 microbiome samples from donors with malnutrition; see Table S1 for a list of all 3,072 TIR domains, including annotations of their proteins of origin, their assigned BPPS groups, and the corresponding human donors and fecal samples).

We subsequently selected 152 TIRs representing 23 different BPPS groups from a survey of (1) >7,000 bacterial proteomes represented in the UniProt database (release 02_2020), (2) 215 phylogenetically diverse human gut bacterial strains that we had cultured, or (3) TIRs that we had identified in the microbiomes described above. Eighteen BPPS groups identified in the developing microbiomes of the Bangladeshi infants and children were represented among these 152 TIRs. Each of these TIR sequences was produced in *E. coli* using an inducible expression vector so that we could conduct an *in vitro* screen of their NADase activities; the products of TIR-domain-mediated NAD metabolism were characterized using a high-performance liquid chromatography (HPLC) assay of lysates prepared from induced (and control non-induced) cells (e.g., Figure 1C). We identified 28 bacterial TIR domains that possessed NADase activity among the 152 screened. These 28 active TIRs, along with eight other bacterial TIR domains that had been reported in the literature (Essuman et al., 2018; Eastman et al., 2021), represent 12 BPPS groups and a phylogenetically diverse group of organisms (Figure S1; Table S2). There are a number of reasons why an assay of this type could produce false-negative results. Therefore, we could not exclude the possibility that some of the TIRs examined could possess enzymatic activity, either as isolated domains or if expressed in the form of their intact parental protein under different conditions.

The *in vitro* assay identified 11 TIR domains that were able to generate variant (v-) forms of cyclic ADP-ribose (cADPR); one form, v-cADPR-x, has been described previously as an *in vitro* product of bacterial TIRs from known mammalian bacterial pathogens (*Acinetobacter baumannii* and *Brucella melitensis*; Essuman et al., 2018), while another, v-cADPR-y, has only been reported in a plant pathogen, *Pseudomonas syringae* (Figures 1C, 1D, and S1; Table S2; Eastman et al., 2021). LC-quadrupole time-of-flight mass spectrometry (LC-QTOF-MS) demonstrated that cADPR, v-cADPR-x, and v-cADPR-y have identical m/z (542.069). However, the three species can be readily resolved by their distinct retention times in the HPLC systems employed in our *in vitro* assay (Figure 1C). Moreover, MS/MS spectra generated by LC-triple quadrupole MS (LC-QqQ-MS) revealed that these two TIR NADase products yielded very similar fragmentation patterns to a cADPR standard (Figure 1E), providing additional evidence that they are variant forms of cADPR.

Based on these results, we assembled a collection of 26 human gut bacterial isolates (Table 1) consisting of type strains as well as strains we had cultured from the fecal microbiota of infants and children living in Bangladesh and Malawi (Blanton et al., 2016; Gehrig et al., 2019); they are prominent members of the human gut microbiota and known colonizers of the intestines of germ-free mice, containing a total of 42 different genes encoding proteins with 44 TIR domains belonging to 14 BPPS groups, including 9 of the top 10 most abundant BPPS groups in our microbiome dataset (Figures 1B and S1). The number of TIR-domain-containing proteins ranged from 0 to 4 per bacterial strain (Table 1); with two exceptions, each of these proteins contained one TIR domain. Twelve of the 44 TIR domains had NADase activity *in vitro* yielding “canonical” products of NAD degradation (nicotinamide, ADPR, and cADPR), as well as the two variant forms of cADPR that have only been detected in *in vitro* assays of TIR enzymatic activity (Tables 1 and S2; Eastman et al., 2021; Essuman et al., 2018; Wan et al., 2019).

**Table 1:** TIR domain content and NADase activities of cultured human gut bacterial isolates

Strain	No. of TIRs	Active TIR domains	ADPR products
*Bacteroides coprophilus* DSM 18228	4	none	N/A
*Bacteroides dorei* DSM 17855	1	none	N/A
*Bacteroides ovatus* ATCC 8483	3	Bovatus_RS22005.TIR_2.1	cADPR
*Bacteroides thetaiotaomicron* 3731	3	AMN69_RS06490.TIR_2.1	cADPR
*Bacteroides thetaiotaomicron* 3731	–	AMN69_RS28245.TIR_2.1	cADPR
*Bacteroides thetaiotaomicron* 7330	2	Btheta7330_RS03065.TIR_2.1	v-cADPR-x
*Bacteroides thetaiotaomicron* 7330	–	Btheta7330_RS23835.TIR_2.1	cADPR
*Bacteroides uniformis* BUAKA3JSW	3	none	N/A
*Bacteroides vulgatus* ATCC 8482	1	none	N/A
*Bacteroides xylanisolvens* XB1A	3	BXY_39700.TIR_2.1	v-cADPR-x
*Bifidobacterium longum* BLJG463	1	none	N/A
*Blautia hansenii* DSM 20583	2	none	N/A
*Clostridium bolteae* ATCC BAA 613	2	CLOBOL_01188.TIR_2.1	ADPR
*Clostridium hathewayi* DSM 13479	0	none	N/A
*Clostridium scindens* ATCC 35704	1	none	N/A
*Clostridium symbiosum* CSTS8243C	1	none	N/A
*Collinsella aerofaciens* ATCC 25986	1	none	N/A
*Coprococcus eutactus* ATCC 27759	4	COPEUT_02740.TIR_2.1	ADPR
*Dorea formicigenerans* ATCC 27755	1	DORFOR_RS09155.TIR_2.1	v-cADPR-y
*Dorea formicigenerans* DFSSTS7063	1	none	N/A
*Enterococcus avium* EASS39	1	none	N/A
*Eubacterium cylindroides* DSM 3983	1	HMPREF0367_01592.TIR_2.1	ADPR
*Proteus penneri* ATCC 35198	1	PROPEN_03896.TIR_2.1	ADPR
*Roseburia intestinalis* L1 82	2	ROSINTL182_07906.TIR_2.1	v-cADPR-x
*Ruminococcus torques* RTSSTS7063	1	none	N/A
*Streptococcus constellatus* SCSS39	2	none	N/A
*Streptococcus pasteurianus* SPSS39	2	none	N/A
*Subdoligranulum variabile* DSM 15176	0	none	N/A

The number of TIR domains identified in each genome, domain IDs, and the ADPR products of TIRs with *in vitro* NADase activity are shown.

### Expression and NAD hydrolase activity of TIR domain proteins in the intestines of gnotobiotic mice

**Figure 2:**
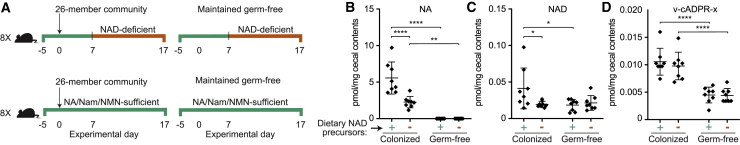
Characterizing the products of NADase-positive TIR domains in the cecal contents of gnotobiotic mice colonized with a 26-member-defined consortium of cultured human gut bacterial taxa and fed NAD precursor-sufficient or deficient diets (A) Design of gnotobiotic mouse experiment. (B–D) Levels of (B) nicotinic acid (NA), (C) NAD, and (D) v-cADPR-x in the cecal contents of germ-free and colonized mice fed the NAD-sufficient or deficient diets. Mean values ± SD are shown. ^∗^p < 0.05; ^∗∗^p < 0.01; ^∗∗∗∗^p < 0.0001 (two-way ANOVA; Tukey’s multiple comparisons test; n = 8 animals/group).

We characterized the *in vivo* expression and biochemical activities of the NADase-positive TIR domains identified in the 26 bacterial strains using gnotobiotic mice. Adult (8-week-old) germ-free C57BL/6J mice were fed a defined diet containing NA, Nam, and nicotinamide mononucleotide (NMN) for 5 days (“NAD-precursor-sufficient” diet in Table S3). Half of the mice were orally gavaged with the consortium of 26 bacterial strains, and the other half were maintained in a germ-free state. One week later, 50% of the mice in the colonized and 50% of the mice in the germ-free groups were switched to a diet that lacked NA, Nam, and NMN (“NAD-precursor-deficient diet” in Table S3) and fed this diet *ad libitum* for 10 days while the other members of each group were continued on the NAD-precursor-containing diet (n = 8 animals/treatment group; four groups; Figure 2A).

The absolute abundances of community members in cecal contents, harvested at the time of euthanasia on experimental day 17, were determined by short read shotgun sequencing of DNA. Read counts were normalized to (1) reads generated from known quantities of two bacterial strains not represented in the community that had been added to cecal samples prior to DNA isolation and (2) the mass of cecal contents. Successful colonization was defined based on reads assigned to five “distractor” genomes that were not represented in or added to the community [cutoff=mean(distractors)+2SD]. Based on this threshold, we determined that all but two of the 26 strains consistently colonized all 16 mice. Successful colonizers included 9 of the 10 strains encoding NADase active TIR domains (Table S4A). Collectively, these nine strains accounted for 39.3% ± 2.2% (mean ± SD) of the total number of genome copies present in cecal contents (community “biomass”). NAD precursor sufficiency versus deficiency in the diet was not accompanied by statistically significant differences in cecal community biomass (p = 0.11; Mann-Whitney U test). *Bacteroides thetaiotaomicron* 3731 was the only strain that exhibited statistically significant diet-associated differences in its absolute abundance (Table S4A); it was present at higher levels in mice fed the NAD-precursor-deficient compared with NAD-sufficient diets (10.6 ± 2.6 × 10^9^ versus 7.2 ± 3.1 × 10^9^ genome equivalents/g cecal contents [mean ± SD], respectively; p = 0.027; Mann-Whitney U-test).

Microbial RNA sequencing (RNA-seq) was used to compare expression of TIR-domain-encoding genes across diet treatments. Of the 10 genes encoding NADase-positive TIR domains present in strains that successfully colonized mice, nine had detectable levels of expression. Among the nine TIRs that they encode, three, generated ADPR *in vitro*, four produced cADPR, and two catalyzed the conversion of NAD to v-cADPR-x (Tables 1 and S2). None of these nine genes exhibited statistically significant differences in their expression as a function of diet in this 26-member community context (Wald test on DESeq2 normalized counts, followed by false discovery rate [FDR] correction; Table S4B).

Targeted mass spectrometry of cecal contents revealed that NA levels were significantly higher in colonized compared with germ-free animals (Table S5A); the effect of diet was mirrored in levels of this NAD precursor, which were significantly higher in colonized animals consuming the precursor-sufficient compared with the precursor-deficient diet (p < 0.001; two-way ANOVA; Tukey’s multiple comparisons test; Figure 2B). NA was not detected in the cecal contents of germ-free mice in either diet context. The concentration of NAD, which, as noted above, is produced in bacteria by *de novo* synthesis from aspartate, was significantly higher in colonized compared with germ-free animals fed the NAD-precursor-sufficient diet (p < 0.03; two-way ANOVA; Tukey’s multiple comparisons test; Figure 2C). Dietary precursor deficiency resulted in a statistically significant decrease in the cecal levels of NAD in mice with the defined bacterial community (p = 0.04), but not in germ-free mice (p = 0.98; two-way ANOVA; Tukey’s multiple comparisons test; Figure 2C). Notably, v-cADPR-x was significantly higher in cecal contents harvested from colonized animals compared with germ-free mice; this was the case in both diet contexts and provided preclinical evidence for its *in vivo* generation by human gut bacteria (p < 0.0001; two-way ANOVA; Tukey’s multiple comparisons test; Figure 2D).

### *Bacteroides xylanisolvens* XB1A produces v-cADPR-x *in vivo*

*Bacteroides thetaiotaomicron* 7330, *Bacteroides xylanisolvens* XB1A, and *Roseburia intestinalis* L1-82 were the only members of the 26-member consortium that contained TIRs that produced v-cADPR-x *in vitro*; *Roseburia intestinalis* L1-82 was present at low abundance, and expression of its v-cADPR-x TIR was not detectable in the cecal meta-transcriptome. These results suggested that one or both *Bacteroides* strains were the source of v-cADPR-x *in vivo*. *Bacteroides thetaiotaomicron* 7330 encodes two proteins containing TIR domains with NADase activity in our *in vitro* assay—one specified by Btheta7330_RS03065 and the other by Btheta7330_RS23835. The TIR domain from Btheta7330_RS03065 produced v-cADPR-x *in vitro*, while the domain from Btheta7330_RS23835 generated Nam and cADPR (Tables 1 and S2). *B. xylanisolvens* XB1A contains three genes whose protein products possess TIR domains. Of the *B. xylanisolvens* XB1A TIR domains tested *in vitro*, only the v-cADPR-x-producing TIR domain found in the protein product of BXY_39700 possessed NADase activity (Tables 1 and S2).

**Figure 3:**
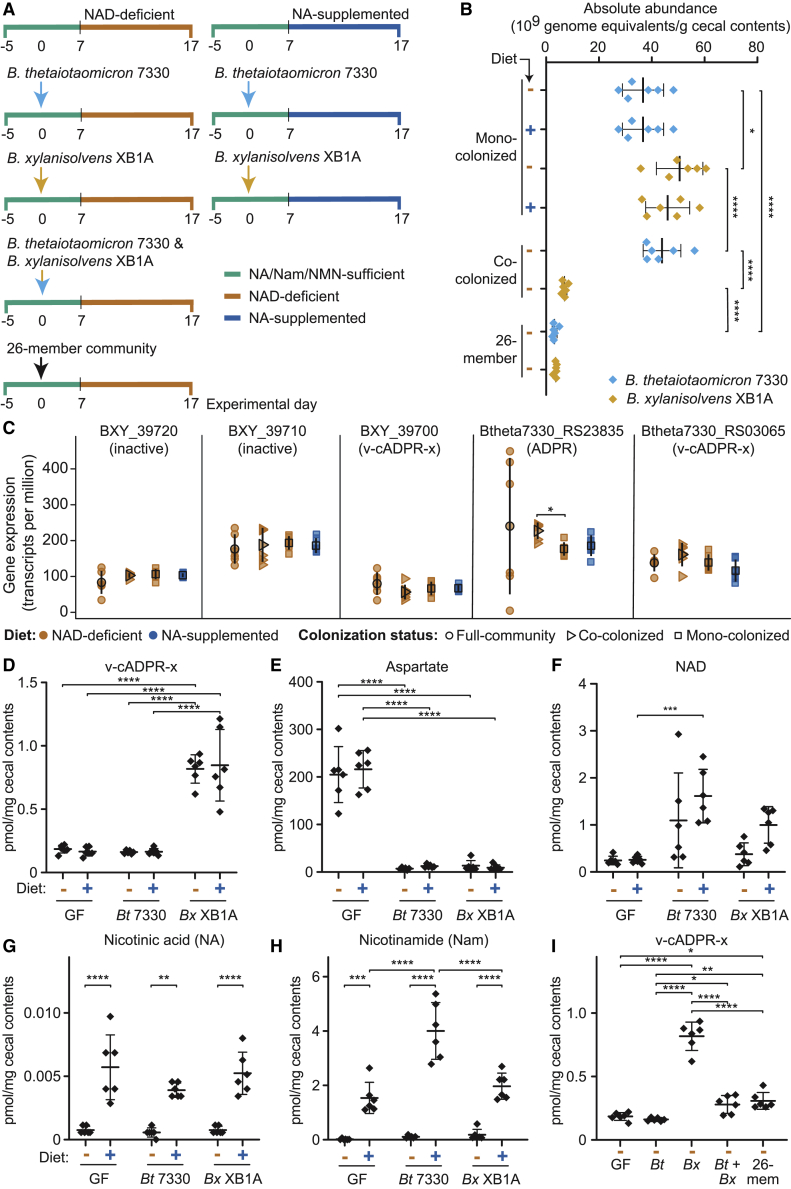
Production of v-cADPR-x in gnotobiotic mice colonized with *Bacteroides xylanisolvens* and/or *Bacteroides thetaiotaomicron* (A) Design of gnotobiotic mouse experiment. (B) Absolute abundance of *B. xylanisolvens* XB1A and *B. thetaiotaomicron* 7330 in cecal contents as a function of diet and community context. Each dot refers to the abundance of the strain within an individual animal. Mean values ± SD are shown. n = 6 mice per treatment group. ^∗^p < 0.05; ^∗∗∗∗^p < 0.0001 (two-way ANOVA; Tukey’s multiple comparisons test). (C) Expression of TIR-domain-encoding genes in *B. xylanisolvens* XB1A and *B. thetaiotaomicron* 7330 as a function of diet and community context (n = 6 mice per treatment group). Mean values ± SD are shown. ^∗^p < 0.05 (DESeq2; Wald test with FDR correction). (D–I) Liquid chromatography-triple quadrupole mass spectrometry (LC-QqQ-MS) of cecal NAD metabolites in germ-free (GF) or mono-colonized animals (D–H) or in animals that had been gavaged with both *B. thetaiotaomicron* 7330 and *B. xylanisolvens* XB1A or the entire 26-member consortium (I). Mean values ± SD are shown. ^∗^p < 0.05; ^∗∗^p < 0.01; ^∗∗∗^p < 0.001; ^∗∗∗∗^p < 0.0001 (two-way ANOVA; Tukey’s post hoc test for comparison of diet and microbial community [D–H] or one-way ANOVA with Tukey’s post hoc test for comparison of community type in mice fed the NAD precursor deficient diet [I]).

A follow-up gnotobiotic mouse experiment was performed to directly determine the origin of v-cADPR-x *in vivo*. Figure 3A describes our experimental design. Groups of 8-week-old, germ-free male C57BL/6J mice were fed the NAD-precursor-sufficient diet for 5 days and then colonized with *Bacteroides thetaiotaomicron* 7330 alone, or with *Bacteroides xylanisolvens* XB1A alone (n = 12 mice/group). After 7 days, mice belonging to a control germ-free arm (n = 12) and the two mono-colonized groups were switched to either the NAD-precursor-deficient diet or an NA-supplemented diet for 10 days (n = 6 mice/diet). As noted above, targeted MS disclosed that cecal levels of NA were significantly higher on the NAD-sufficient compared with NAD-deficient diet in mice colonized with the 26-member consortium (Figure 2C). MS of cecal contents revealed that levels of the NAD precursors NA, Nam, and NMN were very low and not significantly different in germ-free animals fed the NAD-sufficient compared with the NAD-deficient diets (Figure 2C; data not shown). These results led us to surmise that the host was able to efficiently utilize these dietary precursors. Therefore, to ensure that NAD precursors would be readily available to the *Bacteroides* strains, the mono-colonization experiments employed a diet that was supplemented with NA but at a level 50-fold greater than the combined levels of all three NAD precursors (NA, Nam, and NMN) present in the NAD precursor-sufficient diet used in the experiment involving the 26-member community (Table S3).

We found that the absolute abundance of *B. xylanisolvens* XB1A was significantly higher than *B. thetaiotaomicron* 7330 in the cecal contents of mono-colonized animals at the time of euthanasia on experimental day 17 (48.3 ± 8.5 × 10^9^ versus 39.3 ± 7.6 × 10^9^ genome equivalents/g contents; mean ± SD; p = 0.012; two-way ANOVA); this was true in both the NAD-precursor-deficient and NA-supplemented diet contexts (Table S4C; Figure 3B). Microbial RNA-seq demonstrated that Btheta7330_RS03065 in *B. thetaiotaomicron* 7330 and BXY_39700 in *B. xylanisolvens* XB1A (which encode proteins with v-cADPR-x-producing TIR domains) were both expressed in mono-colonized mice. Moreover, levels of their expression were not significantly affected by diet (p = 0.56 and p = 0.88; Wald test on DESeq2 normalized counts, followed by FDR correction; Figure 3C).

LC-QqQ-MS of cecal contents disclosed that, in both diet contexts, v-cADPR-x was present in mice mono-colonized with *Bacteroides xylanisolvens* XB1A; levels were not significantly different in animals consuming one or the other diet (p > 0.9; two-way ANOVA; Tukey’s multiple comparisons test; Figure 3D). Levels of v-cADPR-x were significantly lower in mice harboring *Bacteroides thetaiotaomicron* 7330 or in germ-free animals, irrespective of diet (Figure 3D). In addition, v-cADPR-x was not detectable in plasma recovered from portal vein blood or in plasma isolated from the peripheral blood of mice belonging to any of the four different colonized treatment groups or in the cecal contents, portal vein plasma, or peripheral blood plasma of germ-free controls consuming either diet (data not shown; limits of detection = 18 pmol/mL). The failure to detect v-cADPR-x in the cecal contents of *B. thetaiotaomicron* 7330 mono-colonized mice despite robust expression of Btheta7330_RS03065 raises the possibility of regulation downstream of production of its mRNA. Possible explanations include lack of a stimulus for triggering the multimerization thought to regulate the NADase activities of other TIR domains (Essuman et al., 2018) or differences in the turnover of v-cADPR-x *in vivo*.

LC-QqQ-MS-based measurements of cecal contents revealed that no statistically significant differences existed in aspartic acid, NAD, or NA levels between the two mono-colonized groups of mice in either diet context (Figures 3E–3G; Table S5B). Nam, but not NAD, was significantly elevated in *B. thetaiotaomicron* 7330- compared with *B. xylanisolvens*-colonized animals when they were consuming the NA-supplemented diet (p < 0.0001; two-way ANOVA; Tukey’s multiple comparisons test; Figure 3H). This finding is consistent with the high level of expression of the Btheta7330_RS23835 TIR domain (Figure 3C), which, like all other TIRs with NADase activities that we assayed *in vitro*, generated Nam.

Excessive dietary supply of NA may increase the biosynthetic flux to NAD via NA salvage through the Preiss-Handler pathway. Both strains encode the enzymes necessary for salvage through this pathway. However, neither of these organisms encode PncA, a deamidase that is integral for recycling and salvage of Nam for NAD biogenesis in bacteria (Rodionov et al., 2019). The Nam produced by these active TIR domains thus cannot be internally recycled to NAD and is more likely to be excreted, potentially cross-feeding bacteria that encode PncA.

Other arms in the mouse experiment described in Figure 3A were designed to characterize the effect of community context on production of v-cADPR-x. Mice in these arms were either co-colonized with *B. xylanisolvens* XB1A and *B. thetaiotaomicron* 7330 or gavaged with the complete 26-member community. The absolute abundance of *B. xylanisolvens* was significantly lower in the cecal microbiota of co-colonized mice compared with their mono-colonized counterparts (p < 0.0001; one-way ANOVA; Tukey’s multiple comparisons test; Figure 3B) as were cecal levels of v-cADPR-x (p < 0.0001; one-way ANOVA; Tukey’s multiple comparisons test; Figure 3J). RNA-seq disclosed that (1) the presence of *B. thetaiotaomicron* did not produce significant differences in the levels of expression of *B. xylanisolvens* BXY_39700 and (2), compared with *B. thetaiotaomicron* 7330 mono-colonized animals, the presence of *B. xylanisolvens* in co-colonized animals did not result in a significant change in expression of Btheta7330_RS03065 (Figure 3C). Moreover, the concentration of v-cADPR-x observed in cecal contents was significantly correlated with the absolute abundance of *B. xylanisolvens* XB1A across all community contexts (i.e., mice colonized with either organism alone, both together, and with the complete 26-member consortium; Pearson’s rho = 0.93; p = 5.2 × 10^−21^). Together, the LC-QqQ-MS and microbial RNA-seq results are consistent with *B. xylanisolvens* and its TIR-encoding BXY_39700 gene being the principal source of v-cADPR-x in the cecal contents of mice harboring the 2- and 26-member communities.

### v-cADPR-x TIR domains in the developing gut microbiome

**Figure 4:**
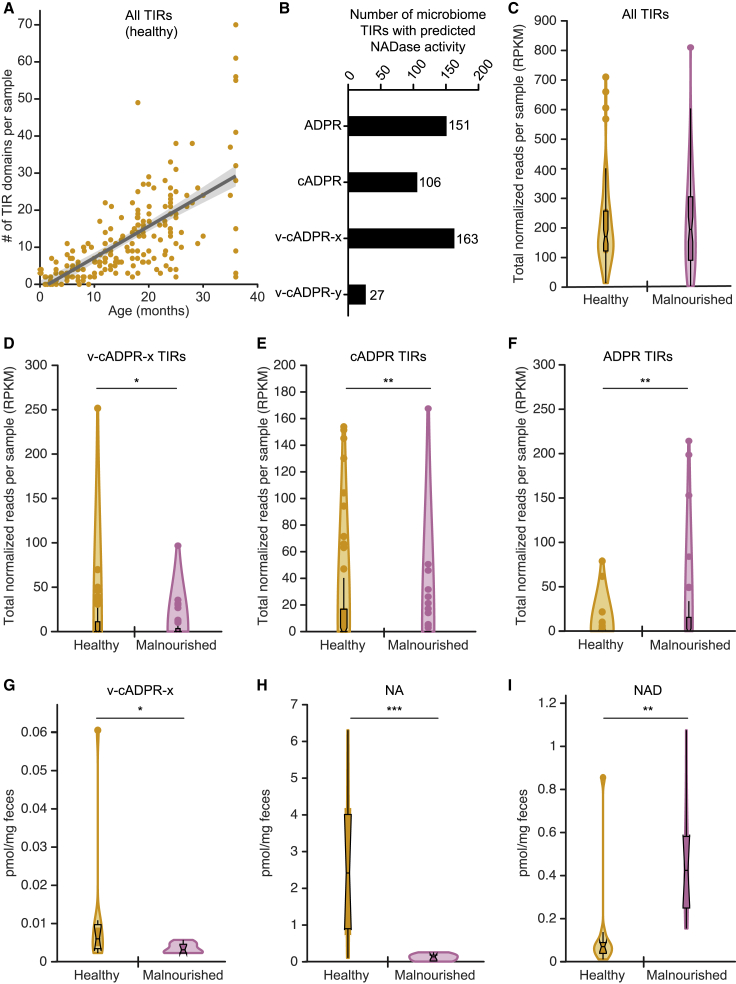
Identification and characterization of v-cADPR-x-producing TIR domains in the fecal microbiomes of healthy Bangladeshi infants and children and those with malnutrition (A) Total number of TIR domains encoded by the fecal microbiomes of healthy Bangladeshi infants and children during the first 3 years of postnatal life. (B) Number of TIR domains identified in the fecal microbiomes of Bangladeshi infants and children (healthy and malnourished) predicted to metabolize NAD to ADPR, cADPR, v-cADPR-x, or v-cADPR-y. (C–F) Violin plots showing the total number of reads, generated from the fecal microbiomes of age-matched infants and children with healthy growth phenotypes or with acute malnutrition (weight-for-length *Z* score [WLZ] < −2), that mapped to all detected TIR-domain-containing genes (C) and genes encoding TIR domains predicted to produce v-cADPR-x (D), cADPR (E), or ADPR (F). Data are normalized for gene length and sequencing depth (n = 82 healthy and 66 SAM donor samples). ^∗^p < 0.05; ^∗∗^ <0.01 (Mann-Whitney U test). (G–I) Levels of v-cADPR-x, nicotinic acid (NA), and NAD were quantified by LC-QqQ-MS of feces collected from members of a healthy birth cohort, or children with acute malnutrition. Violin shape illustrates the distribution of values; the inset box plots indicate median values and interquartile range. ^∗^p < 0.05; ^∗∗^p < 0.01; ^∗∗∗^p < 0.001 (Mann-Whitney U test; n = 10 samples/group).

As noted above, our HMM-based approach identified 3,072 TIR domains distributed across 23 BPPS groups in the proteomes encoded by the microbiomes of 30 serially sampled healthy members of a Bangladeshi birth cohort living in Dhaka and 14 serially sampled children from the same locale who presented with SAM. Turning first to the developing gut microbiomes of the healthy and non-wasted infants and children (weight-for-length [height] *Z* scores no more than two standard deviations below the mean value for a multi-national World Health Organization cohort of infants and children), we identified 2,504 TIRs and determined that TIR domain richness increased significantly during the first 3 postnatal years (β_1_ = 0.75; p = 9.32 × 10^−29^; generalized linear mixed-effects model including a random effect of individual and fixed effects of age and sequencing depth [TIR richness ∼ β_1_(age) + β_2_(reads) + (1|PID)]; Figure 4A).

We subsequently defined the known or putative enzymatic activities of TIR domains identified in the microbiomes of these infants and children based on the relatedness of the TIRs to those characterized *in vitro*. To do so, all of the 3,072 TIRs that are listed in Table S1, as well as all of the 160 TIRs shown in Table S2 that had been assayed *in vitro* (i.e., the 152 that we had selected from our bioinformatic analysis and eight previously reported to have activity), were aligned to an HMM profile for Pfam clan CL0173 (STIR; i.e., the clan that encompasses all of the Pfam families originally used to identify TIR domains in the microbiome dataset). All insertions in sequences aligned to the STIR profile were removed, yielding 116 conserved residues and positions. We then used these positions to calculate pairwise Jukes-Cantor distances (JC) between all TIR domain sequences in the microbiome dataset listed in Table S1 and all sequences listed in Table S2 that had been characterized *in vitro*. We assigned a predicted function to each TIR in the microbiome dataset based on (1) its minimum JC to a TIR with biochemically validated activity (maximum JC allowed = 1.75), (2) ≥98 amino acid residues aligned to the 116 conserved positions, and (3) presence of glutamate at the known catalytic site of TIRs (Essuman et al., 2017). Using this approach, we designated 447 of the 3,072 TIR domains as known or putative NADases. Among these 447 TIRs, the largest number were categorized as v-cADPR-x producers (n = 163 compared with 151 as producers of ADPR, 108 as producers of cADPR, and 27 as producers of v-cADPR-y; Figure 4B; Table S1).

We used this classification method to compare the abundance of TIR domains assigned to different functional classes in fecal microbiome samples collected from the infants and children with acute malnutrition and from those defined as healthy based on serial anthropometry. Given the observed relationship between TIR domain abundance and chronologic age, and the unequal distribution of ages at the time of sampling between the two groups, we subsampled the healthy cohort to compare biospecimens representing comparably aged children who were healthy (n = 82) with those who were malnourished (n = 66). Based on the total number of reads that mapped to TIR-domain-containing ORFs per fecal sample (with data normalized to TIR-containing ORF length and the total number of reads mapping to all ORFs in that sample), we determined that there were no statistically significant differences in overall TIR domain abundance in children with healthy growth phenotypes compared with those with acute malnutrition (Mann-Whitney U test; p = 0.75; Figure 4C; see Table S6 for cumulative read counts per sample). However, TIR domains predicted to produce v-cADPR-x were significantly enriched in the microbiomes of healthy infants and children compared with their counterparts with malnutrition (p = 0.013; Mann-Whitney U-test; Figure 4D), as were those predicted to produce cADPR (p = 0.004; Mann-Whitney U test; Figure 4E). We lacked statistical power to compare the abundance of TIR domains predicted to produce v-cADPR-y, as only 27 were identified across all microbiomes sampled (22 from healthy individuals and five from those with acute malnutrition). In contrast, the cumulative number of normalized reads mapping to TIRs predicted to produce ADPR was significantly elevated in the fecal microbiomes of Bangladeshi children with malnutrition (p = 0.007; Mann-Whitney U test; Figure 4F). These results were consistent across 10,000 bootstrap replicates of the subsampling procedure.

Together, these findings suggested that v-cADPR-x-producing TIR domains may be an informative biomarker of healthy gut microbiome development. Follow-up LC-QqQ-MS assays (Table S5C) showed that v-cADPR-x was significantly elevated in the feces of healthy compared with malnourished members of our study cohort, mirroring the observed discordance in their microbiome-encoded TIR domains predicted to produce this metabolite (p = 0.047; Mann-Whitney U test; Figure 4G). Moreover, across all fecal samples tested, the level of v-cADPR-x was significantly correlated with the abundance of putative v-cADPR-x-producing TIR domains (Pearson’s rho = 0.98; p = 2.87 × 10^−12^), while there was no correlation between the level of TIR domains predicted to produce ADPR and the level of v-cADPR-x (Pearson’s rho = −0.12; p = 0.63). Other possible NAD breakdown products, including ADPR, cADPR, and v-cADPR-y, were not detected with the mass spectrometric conditions we employed. Levels of NA, a metabolite produced from Nam during the salvage and recycling of NAD through the action of the bacterial deamidase, PncA, were significantly higher in the feces of healthy infants and children compared with those who were malnourished (p = 6.7 × 10^−4^; Figure 4H), while NAD was significantly elevated in the feces of those with acute malnutrition (p = 1.9 × 10^−3^; Mann-Whitney U test; Figure 4I).

## Discussion

The importance of NAD and its metabolic products to human systems biology is well established, with knowledge in this area expanding rapidly (Katsyuba et al., 2020). This expanding knowledge base includes information about the contributions of the gut microbiome to NAD biosynthesis and metabolism in health and disease. An example of the latter is the role of a mucus-associated gut bacterium, *Akkermansia muciniphila*, in producing nicotinamide that can ameliorate neurodegeneration in a transgenic mouse model (*Sod1-*Tg) of amyotrophic lateral sclerosis (ALS), with supporting evidence coming from microbiome and metabolite measurements in individuals with ALS and asymptomatic members of the same household (Blacher et al., 2019). Our findings suggest that there is a reduction of TIR NADase activity, specifically v-cADPR-x production, in the gut microbiomes of the sampled Bangladeshi children with acute malnutrition compared with those who were healthy. A corollary is that these children may have decreased substrate availability for their gut bacterial deamidases—enzymes that convert Nam to NA within the intestine and that have been reported to alter the pool of NAD metabolites in extraintestinal sites (e.g., liver and kidney) due to differences in the ability of tissues to utilize deamidated and amidated NAD metabolites (Shats et al., 2020).

Our observations support the notion that an encompassing view of NAD metabolism in the gut should not only consider the well-known conserved pathways for its biosynthesis and biotransformation to canonical products but also the complex “apparatus” of NADases embedded in myriad microbial proteins in the form of TIR domains. The evidence we present in support of this encompassing view reflects the results of computational and experimental approaches. We first used *in silico* methods to identify TIR domains in established databases of bacterial proteomes and in microbiome datasets we generated by shotgun sequencing of fecal DNA from children who were healthy or malnourished. We then determined whether identified TIRs had NADase activity by using *in vitro* expressed TIR domains and mass spectrometric analysis of their metabolic products. NADase activity was observed in TIRs represented in phylogenetically diverse commensal gut bacterial taxa. We subsequently selected a consortium of cultured sequenced human gut bacteria whose genomes encoded proteins containing TIR domains representative of the sequence diversity of TIRs found in the gut microbiome and NADase activities observed *in vitro*; these organisms were introduced into germ-free mice, and expression of their TIR domains and the products of their NADase activities were characterized within the intestine in the context of NAD-precursor-sufficient and deficient diets. NADase active TIR domains from multiple community members were expressed at a high level *in vivo*; v-cADPR-x, a variant of cADPR only known to be produced by TIR enzymes, was detected in colonized, but not germ-free, animals. The results guided our subsequent dissection of which community members produce which products of TIR NADase activity. Identification of TIRs that produce v-cADPR-x *in vitro* and *in vivo*, plus our preclinical evidence suggesting that it is a relatively stable NAD metabolite, provided the rationale for determining whether these findings translated to humans. In a small pilot study, we quantified TIR domains in the developing serially sampled microbiomes of Bangladeshi infants and children with healthy growth phenotypes and of children who presented with severe acute malnutrition. The results obtained from fecal samples collected several years prior to the analysis and maintained at −80°C, indicate a correlation between v-cADPR-x TIR domain abundance, levels of this metabolite, and nutritional status.

v-cADPR-x was originally reported as a product of TIR domains from two pathogenic bacteria, *Acinetobacter baumannii* and *Brucella melitensis* (Essuman et al., 2018), while v-cADPR-y was produced by a TIR domain encoded by the plant pathogen, *Pseudomonas syringae* (Eastman et al., 2021). Analysis of these two metabolites, alongside cADPR, by LC-QTOF-MS and MS/MS fragmentation confirmed that these are likely to be variant forms of cADPR. However, additional structural studies are needed to confirm the cyclization sites of these metabolites.

Our findings emphasize the need to advance this work by generating “metagenome assembled genomes” (MAGs) (i.e., the genomes of bacterial strains represented in the microbiome) from larger numbers of fecal samples collected from more individuals. This effort, combined with microbial RNA-seq, should help expand knowledge of which bacterial strains harbor these v-cADPR-x TIRs, their genomic context, and the conditions under which they are expressed.

The current study sets the stage for subsequent analyses of the biological effects of v-cADPR-x. Children with SAM have an enteropathy that is manifest in part by defects in small intestinal epithelial barrier function and repair (Chama et al., 2019). Given the reported role of Paneth-cell-derived cADPR in an intercellular signaling pathway that influences intestinal stem cell renewal and lineage commitment under conditions of chronic caloric restriction (Yilmaz et al., 2012) and the fact that v-cADPR-x was not detectable in the systemic circulation in our mouse models, one immediate goal could be to delineate its effects on cell lineages within the gut epithelium and in underlying mesenchyme. A comparative study that uses single-nucleus RNA-seq to characterize the effects of v-cADPR-x, v-cADPR-y, and cADPR administered singly and in various combinations to germ-free mice could be a starting point but would require sufficient quantities of these NAD metabolites for a proper dose-response study to be performed over various time intervals. Irrespective of this lack of information about the function of v-cADPR-x, our results emphasize how the developing human gut microbiome harbors a diverse array of TIR domains, not only in terms of their phylogenetic origins but also the NAD metabolic activities that they possess. Moreover, the representation of TIR domains changes during microbiome development. Together, these findings provide a rationale for conducting further studies of how gut microbial community- and host-based systems interact via TIR domains to regulate NAD metabolism in the human holobiont.

### Limitations of the study

As noted above, a major limitation of the current study is that the biological effects of v-cADPR-x have yet to be defined. Substantial quantities of purified cADPR, v-cADPR-x, and v-cADPR-y will be required to study their structures and to conduct comparative *in vivo* analyses of their biological activities.

Our *in vitro* assay used a consistent polypeptide length for all TIR domains tested; this constraint may produce false negatives if critical amino acids fall outside of the region of the domain tested. In addition, our assay was not designed to identify TIR domain enzymatic activities beyond those related to metabolism of NAD.

Finally, our MS-based analysis was performed on a small number of human fecal samples, limiting our ability to comprehensively characterize changes in NAD metabolites during healthy gut microbiome development or in response to nutritional interventions for malnutrition. In addition, we were unable to reliably detect ADPR, cADPR, or v-cADPR-y in these fecal biospecimens; this could reflect, for example, differences in the stability of these metabolites during long-term storage at −80°C or the sensitivity of the LC-QqQ-MS method we employed.

## STAR★Methods

### Key resources table

**Table:** 

Reagent or resource	Source	Identifier
**Bacterial and virus strains**		

T7 Express lysY/I^q^ Competent *Escherichia coli*	New England BioLabs	C3013I
See Table 1 for bacterial strains used in gnotobiotic experiments	N/A	N/A

**Biological samples**		

Human fecal samples	Gehrig et al. (2019)	N/A

**Critical commercial assays**		

Nextera DNA Library Prep Kit	Illumina, Inc.	FC-131-1096
MEGAclear Transcription Clean-Up Kit	Thermo Fisher, Invitrogen	AM1908
Qubit RNA BR Assay Kits	Thermo Fisher, Invitrogen	Q10210
Ribo-Zero rRNA Removal Kit	Illumina, Inc.	MRZB12424
SMARTer Stranded RNA-Seq Kit	Takara Bio Inc.	634839

**Deposited data**		

Sequencing data from gnotobiotic mice (COPRO-Seq, Microbial RNA-Seq)	This paper	PRJEB48101
*Bacteroides uniformis* BUAKA3JSW genome assembly	This paper	PRJEB48101
Shotgun sequencing of human fecal microbiomes	Gehrig et al. (2019)	PRJEB26419
Mass spectrometry data	This paper	ID ST002117

**Experimental models: Organisms/strains**		

Mouse: Gnotobiotic C57BL/6J	N/A	N/A

**Oligonucleotides**		

TIR domain oligonucleotides	GeneBlocks, IDT	See Table S2 for sequences used for *in vitro* assays

**Recombinant DNA**		

pET30a+	Essuman et al. (2017); EMD Biosciences	69909

**Software and algorithms**		

Hmmer v 3.3.1	Hmmer.org	Hmmer.org
Interproscan v 5.48-83.0	Jones et al. (2014)	https://www.ebi.ac.uk/interpro/about/interproscan/
Diamond v 0.9.34	Buchfink et al. (2021)	https://github.com/bbuchfink/diamond
MATLAB R2021b	MathWorks	https://www.mathworks.com/products/matlab.html
Prism Software v 9.0	GraphPad Software	https://www.graphpad.com/scientific-software/prism/
Python v 3.6.5	Python Software Foundation	https://www.python.org
R v 4.1.0	R Core Team (2020)	https://www.r-project.org/
Trimmomatic v 0.36	Bolger et al. (2014)	http://www.usadellab.org/cms/?page=trimmomatic
Bowtie2 v 2.3.4.1	Langmead and Salzberg (2012)	https://github.com/BenLangmead/bowtie2
FLASH v 1.2.11	Magoc and Salzberg (2011)	https://ccb.jhu.edu/software/FLASH/
SPAdes v 3.11.0	Bankevich et al. (2012)	https://github.com/ablab/spades
QUAST v 4.5	Gurevich et al. (2013)	http://quast.sourceforge.net/
Prokka v 1.12	Seemann (2014)	https://github.com/tseemann/prokka
Cutadapt v 1.16	Martin (2011)	https://cutadapt.readthedocs.io/en/stable/
MassHunter	Agilent Technologies	https://www.agilent.com/en/product/software-informatics/mass-spectrometry-software
DESeq2	Love et al. (2014)	https://bioconductor.org/packages/release/bioc/html/DESeq2.html

**Other**		

Defined mouse diets	Dyets, Inc; Table S3 in this paper	D517252, D517253, D517254
Low-fat, plant polysaccharide-rich chow	Envigo	2018S
Pfam-A v 33.1	El-Gebali et al. (2019)	http://pfam.xfam.org/
UniProt v 02_2020	UniProt Consortium	https://www.uniprot.org/
PureCube HiCap StrepTactin MagBeads	Cube Biotech	34225
Nexera X2 HPLC	Shimadzu Europa GmbH	https://www.shimadzu.eu/nexera-x2-0
Agilent 1290 LC	Agilent Technologies	https://www.agilent.com/en/products/liquid-chromatography
Agilent 6470 triple quadrupole mass spectrometer	Agilent Technologies	https://www.agilent.com/en/product/liquid-chromatography-mass-spectrometry-lc-ms
C18 column, 100x3 mm, 2.6 μm	Kinetex Phenomenex	00D-4462-Y0
C18 reversed-phase Atlantis T3 Column, 100Å, 3 μm, 2.1 mm X 150 mm	Waters Corporation	186003719

### Resource availability

#### Lead contact

Further information and requests for resources and reagents should be directed to and will be fulfilled by the lead contact, Jeffrey Gordon (jgordon@wustl.edu).

#### Materials availability

All unique/stable reagents generated in this study are available from the lead contact with a completed Materials Transfer Agreement.

### Experimental model and subject details

#### Human studies

Fecal biospecimens were from completed human studies that are described in an earlier publication (Gehrig et al., 2019). These samples were obtained from male and female infants and children who ranged in age from 0 to 36 months. These studies were approved by the Ethical Review Committee at icddr,b (ClinicalTrials.gov identifiers NCT01889329 and NCT03084731) and written informed consent was obtained from the parent or guardian of the infants and children. Coded biospecimens were shipped under a Materials Transfer Agreement to Washington University (on dry ice) where they were stored at −80°C, along with associated clinical metadata, in a dedicated biospecimen repository with approval from the Washington University IRB.

#### Gnotobiotic mouse experiments

##### Mouse models

All mouse experiments were carried out using protocols approved by the Institutional Animal Care and Use Committee (IACUC) of Washington University in St. Louis. All gnotobiotic animals had a C57BL/6J background, were male and 8 weeks of age at the beginning of the experiments.

### Method details

#### Assembly of *Bacteroides uniformis* BUAKA3JSW genome

Genomes for all but one bacterial strain utilized in this study were previously published (Gehrig et al., 2019) and are publicly available. A library was prepared from gDNA isolated from purified *Bacteroides uniformis* BUAKA3JSW using the Nextera DNA Library Prep Kit (Illumina) and sequencing of the library was performed using an Illumina MiniSeq instrument [2.72 × 10^6^ bi-directional 150 nucleotide reads]. Adapters were removed from reads using trimmomatic (v0.36; Bolger et al., 2014) and reads that aligned to the human reference genome were removed using Bowtie2 (v2.3.4.1; Langmead and Salzberg, 2012). Paired read that overlapped were merged using FLASH (v1.2.11; Magoc and Salzberg, 2011). The genome was assembled using Spades (v3.11.0; Bankevich et al., 2012) and assembly quality was confirmed using Quast (v4.5; Gurevich et al., 2013) [genome length of 4.71 × 10^6^ bp, N50 of 2.37 × 10^5^]. Genomic features were annotated using Prokka (v1.12; Seemann, 2014). Sequencing reads and the assembled genome have been uploaded to ENA (PRJEB48101).

#### Annotation of TIR domains

To identify TIR domain-containing proteins, hidden Markov models (HMMs) for six Pfam domains [DUF1863, SEFIR, TIR-like, TIR, TIR_2, and TIR_3; all members of Pfam clan CL0173 (Pfam-A v33.1, El-Gebali et al., 2019)] were used as input profiles to the *hmmsearch* tool in Hmmer (v3.3.1; http://hmmer.org/). We used these HMMs to search 7,047 reference bacterial proteomes in UniProt (release 02_2020), 215 cultured human gut bacterial taxa, and the dataset of fecal microbiomes sampled from healthy Bangladeshi infants and children and from those with malnutrition. TIR-domain-containing proteins were defined as those with (i) a full-sequence bit score greater than the Pfam-defined noise cutoff for any of the six HMMs, or (ii) containing a statistically significant TIR domain signature as reported by InterProScan (v5.45-80.0; Jones et al., 2014) and encoding at least one domain with a bit score greater than 12. The following InterPro entries were used to define TIR domain signatures via InterProScan: IPR013568, IPR000157, IPR035897, IPR015032, IPR019302, IPR041340.

TIR domain families defined by HMMs often produce significant hits overlapping the same region of a single protein. In these cases, we kept the TIR domain annotation with the highest bit score, alignment length or alignment accuracy (in that order, as defined by Hmmer), for any domains that shared an overlap of at least 33% (Bramley et al., 2020). The probable start and end positions of these domains, as determined by Hmmer (the envelope coordinates), were used to extract individual TIR domain sequences from the proteins. The individual domains were subsequently assigned names based on the locus tag of the gene where they were found, the Pfam model that identified them, and a count based on the number of domains within a given protein that belonged to that family (in cases where a single protein encodes multiple putative TIR domains).

#### *In vitro* NADase assays

Double stranded (ds) DNAs encoding TIR domains were synthesized (GeneBlocks, IDT); they encompassed an average of 138 (minimum = 120, maximum = 143) amino acids starting four amino acids before the position aligning to the profile HMMs or at the start codon (if that codon was located less than four codons before the first aligning position) (Table S2). All sequences were codon optimized for expression in *E coli* K12. dsDNAs were subcloned into the pET30a(+) *E. coli* expression vector with an N-terminal tandem StrepTag and a C-terminal 6× His tag. Recombinant plasmid DNAs were transformed into *E. coli* C3013I (New England Biolabs).

Single isolated colonies were cultured at 30°C overnight in LB medium supplemented with kanamycin (50 μg/mL). Cultures were then diluted 1:20 in fresh LB medium and shaken at 30°C until they reached an OD600 of 0.6–0.8. For induction of TIR expression, isopropyl β-D-1-thiogalactopyranoside (IPTG) was added to a final concentration of 100 μM and the cultures were shaken at 30°C for 1 h.

An HPLC-based assay was used to initially determine whether the TIRs had NADase activity. Aliquots (500 μL) of the culture were pelleted by centrifugation at 3,000 × g for 10 min at 4°C. The supernatant was discarded, and pellets were resuspended in 200 μL of ice-cold PBS. Cells were recovered by centrifugation (3,000 × g for 10 min at 4°C), and 200 μL of 0.5 M perchloric acid was added; the material was suspended by pulse vortexing and then incubated on ice for 10-30 min. The resulting acid extracts were centrifuged at 20,000 × g for 10 min at 4°C; a 150 μL aliquot of the supernatant was combined with 20 μL of 3M potassium carbonate. The neutralized extracts were centrifuged at 20,000 × g for 10 min at 4°C to pellet precipitated salts; 90 μL of the cleared supernatant was then mixed with 10 μL 0.5 M of potassium phosphate buffer. Metabolites were measured by HPLC (Nexera X2) using a C18 column (Kinetex Phenomenex, 100 × 3 mm, 2.6 μm) (Essuman et al., 2018). Metabolites were identified by co-elution with internal standards.

If NADase activity was detected, the assay was repeated to confirm the identity and quantity of the products. Briefly, following 1 h incubation with or without IPTG, cultures were split into three 200 μL replicates for methanol-chloroform extraction. Samples were centrifuged at 3,000 × g for 10 min at 4°C. The resulting pellets were resuspended in 200 μL of ice-cold 0.9% NaCl, centrifuged (3,000 × g for 10 min at 4°C), and the supernatant was discarded. One hundred microliters of ice-cold 50% methanol was added to each pellet prior to sonication. Sonicated samples were frozen at −80°C until use. Samples were thawed on ice, 50 μL of ice-cold chloroform was added, and the mixture was shaken followed by centrifugation (20,000 × g for 10 min at 4°C). Seventy microliters of the methanol fraction were transferred to a tube containing 50 μL ice-cold chloroform; the mixture was pulse vortexed and centrifuged to recover the methanol fraction. Fifty microliters of the final methanol extract were dried (SpeedVac) and stored at −80°C. Samples were subsequently resuspended in 50 μL of 5 mM ammonium formate. Following centrifugation (20,000 × *g* for 10 min at 4°C), a 10 μL aliquot of the supernatant was injected into C18 reverse phase column (Atlantis T3, 2.1 × 150 mm, 3 μm; Waters) linked to an HPLC system (Agilent 1290 LC) [flow rate of 0.15 mL/min using 5 mM ammonium formate for mobile phase A and 100% methanol for mobile phase B]. Metabolites were eluted with gradients of 0–10 minutes, 0–70% B; 10–15 minutes, 70% B; and 16–20 minutes, 0% B. Metabolites were detected with a triple quadrupole mass spectrometer (Agilent 6470) under positive ESI multiple reaction monitoring (MRM) using parameters for identifying NAD [precursor m/z = 664.11, product m/z = 428.04, fragmentation (F) = 160 V, collision (C) = 22 V and cell acceleration (CA) = 7 V]. Serial dilutions of internal standards in 5 mM ammonium formate were used to generate a standard curve. Metabolites were quantified by MassHunter (Agilent).

The NADase activities of purified TIR domains and TIR domain-encoding proteins were also characterized (e.g., Figure 1B inset). To generate purified protein, overnight cultures of *E. coli* C3013I harboring recombinant pET30a(+)-TIR constructs described above were diluted 1:100 in 100 mL LB supplemented with 50 μg/mL kanamycin. Once cultures reached an OD600 of ∼0.6, IPTG was added (100 μM). Cells were harvested from 50 mL cultures 3 h after induction, and Strep-tagged proteins were purified using 200 μL HiCap Streptactin Mag Beads (Cube Biotech). Ten μL of purified bead-bound proteins were tested for NADase activity in a 50 μL reaction mixture containing HEPES (25 mM) and 10 μM NAD substrate.

#### Annotation of TIR domains in human microbiome datasets

TIR domains were identified and annotated as described above, and their sequences were extracted in the same manner as described for the *in vitro* NADase assay. An HMM profile (model length = 116) was generated from the multiple sequence alignment of clan CL0173 obtained from the online Pfam database (Pfam-A v33.1, El-Gebali et al., 2019) using the *hmmbuild* tool contained within Hmmer (v3.3.1; http://hmmer.org/). Sequences were aligned to the HMM profile using the *hmmprofalign* tool within Matlab, and then subset to include only residues at positions that aligned with the HMM profile (i.e., insertions were removed). Sequences that had gaps (missing residues) in >15% of these 116 positions were removed from downstream analyses. This trimmed and filtered alignment was used to calculate the pairwise Jukes-Cantor distance between all pairs of sequences that remained. TIR domains identified in the microbiome datasets were assigned a predicted function based on the activity observed for the most similar (lowest Jukes-Cantor distance) TIR domains tested *in vitro*, with a maximum Jukes-Cantor distance of 1.75. [The pairwise distance between active TIR domains was significantly lower than the pairwise distance between active and inactive TIR domains (p = 2.65 × 10^−57^; one-way ANOVA)]. Additionally, sequences were required to encode a glutamate at the known catalytic site of TIRs (position 73 in the CL0173 alignment) in order to be classified as active (Essuman et al., 2018).

#### Gnotobiotic mouse experiments

Diets were produced by Dyets, Inc (Bethlehem, PA); they were modified versions of the AIN-93G Purified Rodent Diet (Dyets; catalog number 110700) where the amount of cornstarch was altered to accommodate addition or removal of NAD precursors to maintain levels of all other micronutrients, and most macronutrients (Table S3). The NAD precursor-sufficient diet had NMN and Nam added in amounts that were equimolar to the level of NA in AIN-93G. The NAD-deficient diet contained no NAD-precursors. The NA-supplemented diet contained 55.5 times the molar amount of the combined NAD precursors in the NAD-sufficient diet (Feng et al., 2020). All diets were sterilized by gamma irradiation (30-50 kGy; Sterigenics, Rockaway, NJ). Sterility was confirmed by culturing the diets at 37°C in TYG medium under aerobic and anaerobic (75% N_2_, 20% CO_2_, 5% H_2_) conditions.

Germ-free C57BL/6J mice were reared in plastic flexible film gnotobiotic isolators (Class Biologically Clean) at 23°C under a strict 12-h light cycle (lights on at 0600h, off at 1800h). Animals were weaned onto an autoclaved, low-fat, plant polysaccharide-rich chow (catalog number 2018S, Envigo) that was administered *ad libitum*. Five days prior to colonization, 8-week-old male mice were switched to the NAD precursor-sufficient diet; 2 days later, they were either placed on the NAD precursor-deficient diet, or the NA-supplemented diet, or were continued on the NAD precursor-sufficient diet. All bacterial strains that were introduced to gnotobiotic mice are listed in Table 1; strain identity was confirmed by sequencing of full-length 16S rDNA. Equivalent numbers of bacterial cells (based on OD_600_ measurements), recovered from monocultures grown to stationary phase in LYBHI medium under anaerobic conditions, were introduced into germ-free mice using a plastic-tipped oral gavage needle (Fisher); mice were either mono-colonized or gavaged with mixtures of 2 or 26 bacterial strains (volume of gavage: 200 μL). Animals were maintained in separate gnotobiotic isolators, each dedicated to mice colonized with the same bacterial consortium (n = 3 or 4 animals/cage). Animals received their diets *ad libitum*. Cages contained autoclaved paper ‘shepherd shacks’ to facilitate their natural nesting behaviors and to provide environmental enrichment. Pre-colonization fecal samples were collected to verify the germ-free status of the mice using both culture-based and culture-independent (16S rRNA-based) assays.

#### Community profiling by sequencing (COPRO-Seq)

Short-read COmmunity PROfiling by Sequencing (COPRO-Seq, McNulty et al., 2013) was used to define the absolute abundance of bacterial strains in cecal samples obtained from colonized mice. For absolute abundance determination, known quantities of two spike-in organisms (*Agrobacterium radiobacter* DSM 30147 and *Alicyclobacillus acidiphilus* DSM 14558) were added to each weighed, frozen specimen of cecal contents (Stämmler et al., 2016; Wolf et al., 2019). DNA was isolated from the pellets by adding 500 μL of extraction buffer [200 mM Tris (pH 8), 200 mM NaCl, 20 mM EDTA], 210 μL of 20% SDS, and 500 μL of 0.1 mm diameter zirconia beads, followed by treatment with a BioSpec bead beater for 4 min, addition of 500 μL phenol:chloroform:isoamyl alcohol (25:24:1), and precipitation of nucleic acids with isopropanol. Libraries were prepared using the Nextera DNA Library Prep Kit (Illumina) and combinations of custom barcoded primers (Adey et al., 2010). Multiplex sequencing of the libraries was performed using Illumina MiniSeq and/or NextSeq instruments [uni-directional 75 nucleotide reads; 4.33 × 10^6^ ± 1.99 × 10^6^ reads/sample (minimum of 2.76 × 10^6^ reads)]. Reads were mapped onto the sequenced genomes of consortium members using an analytic pipeline described in Gehrig et al. (2019), as well as to the genomes of the two spike-in bacteria, and five ‘distractor’ genomes belonging to bacteria that were not present in the samples (*Lactobacillus ruminis* ATCC 27782, *Megasphaera elsdenii* DSM 20460, *Olsenella uli* DSM 7084, *Pasteurella multocida* subsp. *multocida* str. 3480 and *Staphylococcus saprophyticus* subsp. *saprophyticus* ATCC 15305). The absolute abundance of each community member was calculated by multiplying the normalized counts of that member with the abundances of the spike-in and dividing by the measured weight of the sample of cecal contents (Stämmler et al., 2016). A strain was considered to be a successful colonizer if its absolute abundance was greater than two standard deviations above the mean absolute abundance of the distractor genomes in a given sample.

#### Microbial RNA-Seq

Snap-frozen aliquots of cecal contents were stored at −80°C. Cecal samples were thawed on ice and reagents were added in the following order: (i) 250 μL of acid-washed glass beads (212-300 μm diameter; MilliporeSigma; catalog number G1277), (ii) 500 μL of Buffer B (200 mM NaCl, 20 mM EDTA), (iii) 210 μL of 20% SDS, and (iv) 500 μL of a mixture of phenol:chloroform:isoamyl alcohol (125:24:1, pH 4.5; ThermoFisher). The mixtures were homogenized in a bead beater (Biospec) for 4 min at room temperature and centrifuged at 8,000 × g for 10 min at 4°C. An aliquot (450 μL) of the aqueous phase was transferred to a new tube on ice and RNA was isolated according to a protocol described in a previous publication (Hibberd et al., 2017). The integrity of total RNA was assessed using a Bioanalyzer (Agilent). Genomic DNA (gDNA) was eliminated by DNase treatment and the samples were purified using the MEGAclear Transcription Clean-Up Kit (ThermoFisher). The absence of gDNA contamination was verified using PCR primers directed against variable region 4 of the bacterial 16S rRNA gene. gDNA-free RNA was quantified using the Qubit RNA BR Assay Kit (Invitrogen) and 1 μg was subjected to ribosomal RNA depletion using the Ribo-Zero (Epidemiology/Bacteria) kit (Illumina) followed by ethanol precipitation. dsDNA and dual-indexed Illumina libraries were prepared using the SMARTer Stranded RNA-Seq kit (Takara Bio USA). Libraries were first sequenced on an Illumina MiniSeq instrument, generating 75-nt reads. Read counts were used to balance the pool and deeper coverage was obtained using an Illumina NextSeq platform [70-nt unidirectional reads; 2.97 ± 1.4 × 10^6^ reads/sample (mean ± SD); n = 51 samples)]. Fluorescence was not measured from the first five cycles because the library preparation introduces three non-templated deoxyguanines that would terminate the sequencing run if imaged. Sequences were trimmed using Cutadapt (v. 1.16 Martin, 2011) to remove adapters, low-quality ends (quality cutoff = 25), and the first three bases of the 75-nt reads generated by MiniSeq. Quality control of the trimmed, demultiplexed, sequences was conducted using FastQC and reads were mapped to the genomes of community members after combining reads generated by the MiniSeq and NextSeq instruments. Raw counts were normalized using library sizes and gene lengths to transcripts per kilobase million (TPM) counts. The resulting dataset was then imported into R, and DESeq2 (Love et al., 2014) was used to identify differentially expressed, TIR domain-encoding genes using a DESeq2 significance threshold of α = 0.1 and raw read counts as input.

#### Mass spectrometry of cecal NAD-related metabolites

Methods used for LC-QqQ-MS-based quantification of these metabolites were similar to those used in the *E. coli* based TIR expression assay described above, with the following modifications. Cecal contents were collected on 10 μL plastic inoculation loops at the time of sacrifice, snap frozen in liquid nitrogen, and stored at −80°C. Aliquots were kept frozen, removed from the loops and weighed. 10 μL of ice-cold methanol was added per mg of sample, which was then thawed on ice. The mixture was subsequently shaken for two minutes in a BioSpec bead beater. Samples were then centrifuged for 10 min at 3,000 × g at 4°C; a 150 μL aliquot of the supernatant was transferred to a new tube containing 150 μL ice-cold chloroform, then mixed by pulse vortexing and centrifuged for 10 min at 3,000 × g at 4°C. One hundred microliters of the methanol fraction was transferred to a new tube containing 100 μL of ice-cold chloroform, mixed by pulse-vortexing, and centrifuged for 10 min at 3,000 × g at 4°C. 70 μL was transferred to a new tube and lyophilized in a Labconco SpeedVac. Samples were resuspended in 40 μL of ammonium formate. At least two samples per treatment group were split into two aliquots, and purified v-cADPR-x was spiked into one of the aliquots. Following centrifugation (20,000 × *g* for 10 min at 4°C), 10 μL of the supernatant was injected into a C18 reverse phase column (Atlantis T3, 2.1 × 150 mm, 3 μm; Waters). The spiked-in v-cADPR-x was used to determine the retention time of this metabolite across biological matrices and experiments.

### Quantification and statistical analysis

Details about the statistical methods employed can be found in the main text and in figure legends. COPRO-Seq and mass spectrometric data for gnotobiotic mouse experiments are expressed as mean values ± standard deviations (SD) with the number of independent biological replicates indicated in the figure legends. Two-way ANOVA followed by Tukey’s multiple comparisons test was used to analyze mass spectrometric data for experiments testing multiple microbial communities and diets. One-way ANOVA followed by Tukey’s multiple comparisons test was used for analyses comparing metabolite levels in mice consuming the same diet colonized with different microbial communities. The non-parametric Mann-Whitney U test was used to compare the abundance of individual strains within the 26-member community between mice fed the NAD-precursor deficient- or sufficient-diet (results shown in Table S4A). To compare relative levels of expression of genes with in a single bacterial strain, the RNA-seq data from gnotobiotic mouse experiments shown in Figures S4B and 3C were normalized based on library size and gene length to transcripts per kilobase million (TPM) and the results shown as mean values ± SD. Analysis of differential gene expression was performed using DESeq2 with p-values determined by applying the Wald test with FDR correction to DESeq2 normalized counts. Human microbiome shotgun sequencing and LC-QqQ-MS data were analyzed with the non-parametric Mann-Whitney U test as they were not normally distributed. Data were processed and analyzed using Bash, Prism Software 9.0 (GraphPad), MATLAB R2021b (Mathworks), R (v4.1.0), and Python (v 3.6.5).

### Additional resources

This work involves data and biospecimens from clinical trials. The clinical registry numbers and associated links are as follows: ClinicalTrials.gov identifiers NCT01889329 (https://clinicaltrials.gov/ct2/show/NCT01889329?term=NCT01889329&draw=2&rank=1) and NCT03084731 (https://clinicaltrials.gov/ct2/show/NCT03084731?term=NCT03084731&draw=2&rank=1).

## Data Availability

• COPRO-Seq and microbial RNA-Seq datasets generated from the intestinal contents of gnotobiotic mice plus shotgun sequencing datasets of the *Bacteroides uniformis* BUAKA3JSW genome have been deposited to European Nucleotide Archive (ENA) and are publicly available as of the date of publication. Accession numbers are listed in the key resources table. • Mass spectrometry data have been deposited in the National Metabolomics Data Repository and are publicly available as of the date of publication. Accession numbers are listed in the key resources table. • This paper analyzes existing, publicly available data; links to the datasets used are listed in the key resources table. • This paper does not report original code. • Any additional information required to reanalyze the data reported in this paper is available from the lead contact upon request.

## Supplemental information

Document S1. Figures S1 and S2

Table S1. List of 3,072 TIR domains identified in 278 fecal microbiome samples obtained from 30 healthy Bangladeshi children and 14 children with acute malnutrition, related to Figures 1 and 4

Table S2. Results of *in vitro* NADase assays for 160 TIR domains, related to Figure 1 Activities, including major and minor products, are shown.

Table S3. Composition of diets used in gnotobiotic mouse experiments, related to Figures 2 and 3

Table S4. Abundance of members of bacterial consortia measured in cecal contents of gnotobiotic mice and expression of TIR genes with *in vitro* NADase activity, related to Figures 2 and 3 (A) Absolute abundances of bacterial strains in cecal contents harvested from animals studied in the experiment shown in Figure 2A. (B) Microbial RNA-Seq analysis, performed using the cecal contents of mice described in (A), of the expression of genes encoding TIR domains with *in vitro* NADase activity as a function of dietary NAD-precursor availability. (C) Absolute abundances of bacterial strains in cecal contents of mice characterized in the experiment described in Figure 3A.

Table S5. LC-QqQ-MS measurement of NAD metabolites in gnotobiotic mice and humans, related to Figures 2, 3, and 4 (A) Cecal NAD metabolites in animals gavaged with the entire 26-member consortium or maintained germ-free (GF). Cecal contents were collected on day 17 of the experiment shown in Figure 2A. Data correspond to that shown in Figures 2B–2D. (B) Cecal NAD metabolites in germ-free mice, mono-colonized mice, animals gavaged with both *B. thetaiotaomicron* 7330 and *B. xylanisolvens* XB1A or mice harboring the 26-member bacterial consortium. Cecal contents were collected on day 17 of the experiment described in Figure 3A. Data correspond to Figures 3D–3I. (C) NAD metabolites present in the feces of Bangladeshi infants and children that were healthy or malnourished.

Table S6. Abundance of TIR domains and their predicted NADase activities in sampled fecal microbiomes, related to Figure 4 The cumulative normalized count of reads (RPKM) within each sampled microbiome mapped to (i) all TIR domain-encoding genes, or TIR domains predicted to produce (ii) ADPR, (iii) cADPR, (iv) v-cADPR-x and (v) v-cADPR-y.

Document S8. Article plus supplemental information
