# Supplementary material for: Products of gut microbial Toll/interleukin-1 receptor domain NADase activities in gnotobiotic mice and Bangladeshi children with malnutrition
Source: Cell Rep. 2022 Apr 26;39(4):110738. doi: 10.1016/j.celrep.2022.110738 (PMC9092222; doi:10.1016/j.celrep.2022.110738)
Supplement: Document S1. Figures S1 and S2 [file mmc1.pdf]

Cell Reports, Volume *39*

Supplemental information

Products of gut microbial Toll/interleukin-1 receptor domain NADase activities in gnotobiotic mice and Bangladeshi children with malnutrition

James S. Weagley, Mark Zaydman, Siddarth Venkatesh, Yo Sasaki, Neha Damaraju, Alex Yenkin, William Buchser, Dmitry A. Rodionov, Andrei Osterman, Tahmeed Ahmed, Michael J. Barratt, Aaron DiAntonio, Jeffrey Milbrandt, and Jeffrey I. Gordon

Weagley et al., Fig. S1

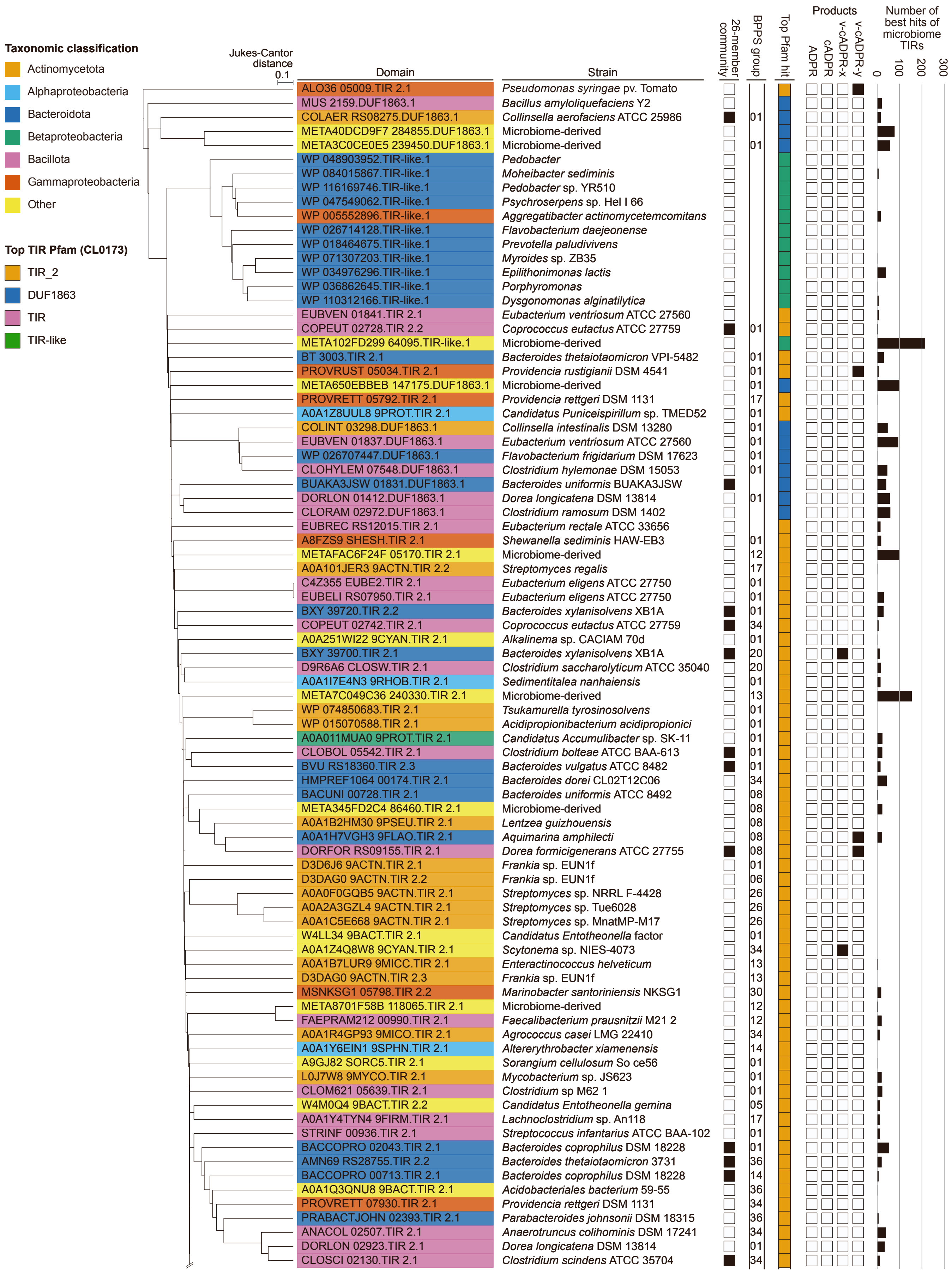

Figure S1. Characterization of *in vitro* NADase activity in TIR domains identified in 116 cultured bacterial strains and in the gut microbiomes of Bangladeshi children (Related to Figure 1). Tree depicting the relationship between all sequences tested *in vitro*. The tree was constructed from pairwise Jukes-Cantor distances calculated across the 116 positions represented in the CL0173 HMM profile. Displayed are the BPPS groups and Pfam domains to which TIR domains were assigned, the gene and organism where the domain was identified, and the results obtained from an *in vitro, E. coli*-based assay of the NADase activities of these TIRs. TIR domains encoded by the 26-member community used to colonize gnotobiotic are noted by the black-filled boxes. The right most column shows the number of TIRs in sampled microbiomes that were assigned a predicted function based on their similarity to the domain tested *in vitro.* The angled double-hash mark indicates the break point connecting the tree in this figure to the tree in Figure S2.

Weagley et al., Fig. S2

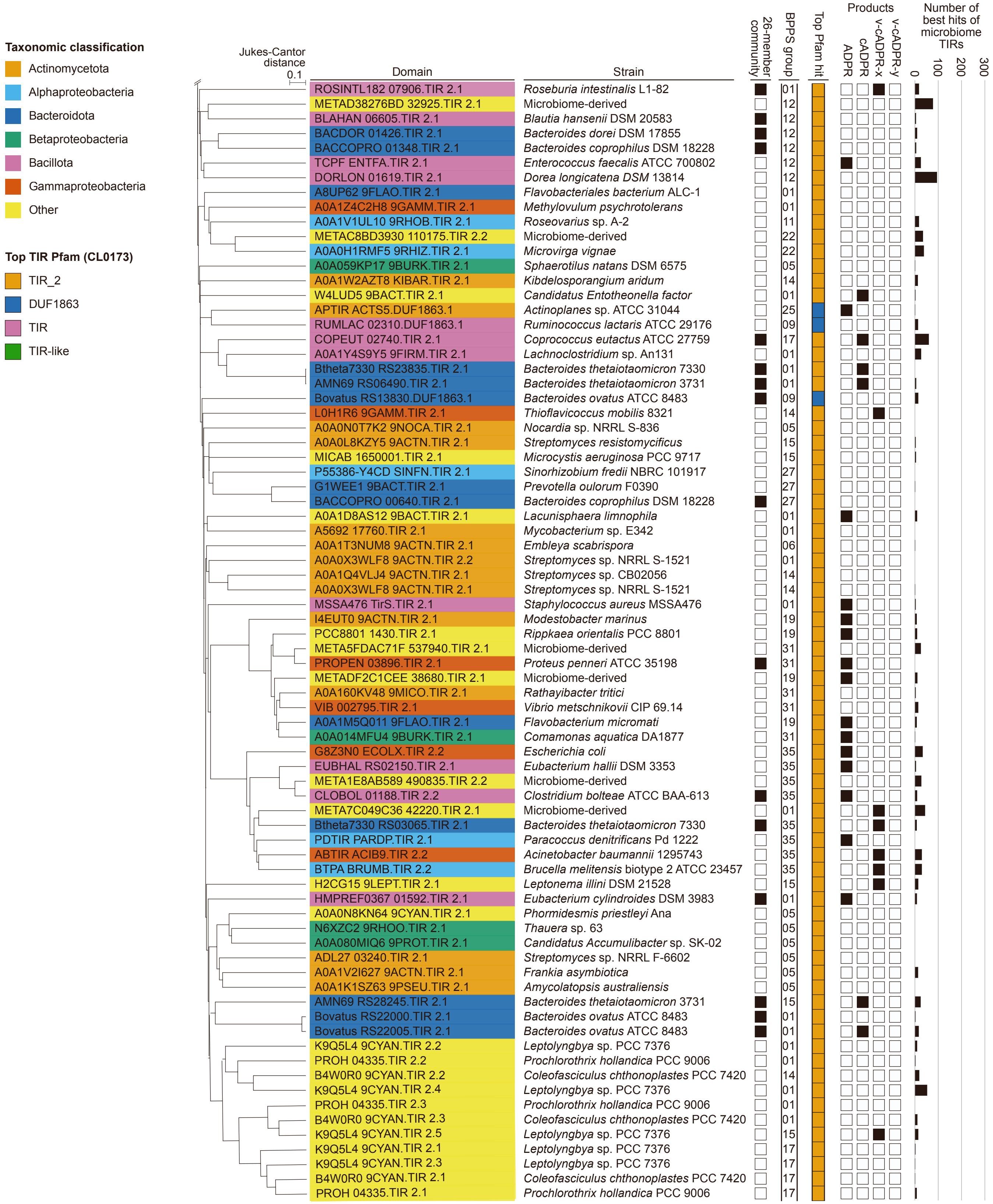

Figure S2. Characterization of *in vitro* NADase activity in TIR domains identified in 116 cultured bacterial strains and in the gut microbiomes of Bangladeshi children (Related to Figure 1). See legend to Figure S1. The angled double-hash mark indicates the break point connecting the tree in this figure to the tree in Figure S1.
